# Clinical Validity of a Machine Learning Decision Support System for Early Detection of Hepatitis B Virus: A Binational External Validation Study

**DOI:** 10.3390/v15081735

**Published:** 2023-08-14

**Authors:** Busayo I. Ajuwon, Alice Richardson, Katrina Roper, Brett A. Lidbury

**Affiliations:** 1 National Centre for Epidemiology and Population Health, ANU College of Health and Medicine, The Australian National University, Acton, Canberra, ACT 2601, Australia; katrina.roper@anu.edu.au (K.R.); brett.lidbury@anu.edu.au (B.A.L.); 2 Department of Biosciences and Biotechnology, Faculty of Pure and Applied Sciences, Kwara State University, Malete 241103, Nigeria; 3 Statistical Support Network, The Australian National University, Acton, Canberra, ACT 2601, Australia; alice.richardson@anu.edu.au

**Keywords:** HepB LiveTest, hepatitis B virus, machine learning decision support system, clinical validity, external validation

## Abstract

HepB LiveTest is a machine learning decision support system developed for the early detection of hepatitis B virus (HBV). However, there is a lack of evidence on its generalisability. In this study, we aimed to externally assess the clinical validity and portability of HepB LiveTest in predicting HBV infection among independent patient cohorts from Nigeria and Australia. The performance of HepB LiveTest was evaluated by constructing receiver operating characteristic curves and estimating the area under the curve. Delong’s method was used to estimate the 95% confidence interval (CI) of the area under the receiver-operating characteristic curve (AUROC). Compared to the Australian cohort, patients in the derivation cohort of HepB LiveTest and the hospital-based Nigerian cohort were younger (mean age, 45.5 years vs. 38.8 years vs. 40.8 years, respectively; *p* < 0.001) and had a higher incidence of HBV infection (1.9% vs. 69.4% vs. 57.3%). In the hospital-based Nigerian cohort, HepB LiveTest performed optimally with an AUROC of 0.94 (95% CI, 0.91–0.97). The model provided tailored predictions that ensured most cases of HBV infection did not go undetected. However, its discriminatory measure dropped to 0.60 (95% CI, 0.56–0.64) in the Australian cohort. These findings indicate that HepB LiveTest exhibits adequate cross-site transportability and clinical validity in the hospital-based Nigerian patient cohort but shows limited performance in the Australian cohort. Whilst HepB LiveTest holds promise for reducing HBV prevalence in underserved populations, caution is warranted when implementing the model in older populations, particularly in regions with low incidence of HBV infection.

## 1. Introduction

Hepatitis B virus (HBV) is a significant public health concern, causing liver infection and leading to substantial morbidity and mortality worldwide. With over 296 million people living with HBV globally, 90% of infected individuals are unaware of their infection status, missing out on essential clinical care [1,2]. In 2019, HBV-related deaths reached a staggering 820,000 [1], emphasising the urgent need for an innovative approach to enhance early detection and stop transmission within populations. Addressing this global health challenge necessitates a multifaceted strategy that integrates advances in digital innovations and population health. By leveraging this interdisciplinary approach, healthcare professionals can be empowered to detect HBV infections earlier and provide timely linkage to care.

Our prior investigation into HBV infection levels in Nigeria revealed a prevalence of 9.5% [3], highlighting the substantial burden of HBV in the West African country. Even countries with lower prevalence, such as Australia, grapple with disproportionately high infection rates among vulnerable and marginalised communities. These include individuals from diverse ethnic backgrounds, Indigenous people such as the Aboriginal and Torres Strait Islander populations, people who use drugs, and incarcerated individuals [4]. Early detection of HBV is therefore critical, as delayed diagnosis can lead to severe and life-threatening clinical complications of liver damage and end-stage hepatocellular carcinoma.

To support the World Health Organization’s goal of eliminating viral hepatitis by 2030 [5], there has been growing interest in developing machine learning models that integrate routine pathology data to predict HBV infections earlier [6,7,8], considering that specialised HBV tests are expensive and not readily available in resource-constrained settings. These prediction models can serve as decision support systems, enhancing patient care and providing actionable insights to clinicians in routine clinical practice.

In a recent study, we developed HepB LiveTest, a machine learning decision support system for early detection of HBV infection based on routine blood test data, including hepatitis B surface antigen (HBsAg) immunoassay results [9]. The model learned from patient data, identified patterns, and intelligently predicted a patient’s HBV infection status with a discrimination threshold of 90%. This innovative approach holds immense potential in revolutionising the landscape of HBV diagnosis and patient care, enabling timely interventions for improved health outcomes. Given the potential impact of the machine learning decision support system, we sought to externally validate its generalisability and robustness in independent patient cohorts from different settings and populations. This is an important step towards establishing the cross-site transportability and robustness of HepB LiveTest across diverse settings and populations, thus contributing to its seamless integration into routine clinical workflow.

Conducting external validation separately from the model development has been recommended to ensure methodological rigor, reduce biases, and increase the transparency of performance evaluation and generalisability in new and diverse patient populations. This approach enhances the credibility and practical utility of clinical prediction models in a real-world setting [10,11,12,13]. Unfortunately, most prediction research only focuses on model development and many clinical prediction models lack multi-site testing [14,15,16], leading to discrepancies between locally reported performance and cross-site generalisability. This often leads to a plethora of proposed models, with little evidence about the extent of their generalisability and under what circumstances. Confusion then ensues, promising models are often quickly forgotten [17] and, of more concern, many models may be used or advocated without appropriate evaluation of their cross-site transportability. Therefore, external validation is crucial in assessing a model’s performance beyond its development dataset, considering that covariate–outcome relationships may vary between patient populations and settings.

Predictor and outcome measurements may vary for various reasons, thus distorting the performance of a prediction model. Variability in measurements can arise from differences in equipment specifications, timing of data collection, subjectivity in interpretation, and nuances in biomarker quantification. These factors can introduce heterogeneity in predictive modelling studies, based on electronic health records. Such variations in measurement procedures may significantly impact the discriminative performance of the prediction model and also compromise its clinical validity in different patient populations and settings—and seemingly “better” measurements at validation may also not necessarily lead to improved model performance [18,19]. This underscores the need for comprehensive and robust validation of prediction models in different population settings.

The main objective of this study is to independently validate HepB LiveTest in two external patient cohorts from Nigeria and Australia and evaluate the case-mix variability on performance drift. This geographic validation is critical in determining whether HepB LiveTest accurately predicts HBV infection in patients from diverse populations/settings, providing insights into the model’s generalisability and potential clinical utility. By evaluating the performance of HepB LiveTest in independent patient cohorts, we aim to contribute valuable evidence to inform the adoption and appropriate use of this machine learning decision support system for early detection of HBV infection.

## 2. Methods

The study protocol was approved by the Institutional Review Board of the University of Ilorin Teaching Hospital (ERC PAN/2020/06/0022) and the Human Research Ethics Committee of the Australian National University (2019/803) as minimal-risk research that used retrospective patient data collected from routine clinical care and, as such, the requirements for informed consent were waived.

The study was reported in accordance with the Transparent Reporting of a Multivariable Prediction Model for Individual Prognosis or Diagnosis (TRIPOD) guideline for prediction model validation [10].

### 2.1. Cohort Selection/External Validation Dataset

To assess the clinical validity and cross-site transportability of HepB LiveTest model, it was externally validated in independent Australian and Nigerian patient cohorts, with datasets collected from the Sullivan Nicolaides Pathology (SNP Taringa, Queensland, Australia) and the University of Ilorin Teaching Hospital (UITH, Kwara State, Nigeria), respectively, across two different time periods.

SNP is Australia’s largest private pathology referral laboratory, regarded for its expertise in routine pathology testing. It delivers comprehensive laboratory services to hospitals in Queensland, northern New South Wales, and the Northern Territory, with the central laboratory in Brisbane designed to foster interdisciplinary collaboration between specialist pathologists and clinical scientists, while UITH is one of the major Federal Teaching Hospitals in Nigeria, located within the North Central Geopolitical Zone of Nigeria at latitude 8°30′ N and longitude 4°33′ E. The hospital provides care to a large number of patients from Kwara State and equally serves other neighbouring states, including Oyo, Niger, Kogi, and Ekiti.

The two validation datasets from SNP and UITH included patients suspected of HBV infection and who had undergone HBsAg immunoassay testing. Patient samples to produce the SNP validation dataset were collected between 1 June 2011 and 31 May 2012, and samples to produce the UITH validation dataset were collected between 1 April 2018 and July 2021. All patient records were anonymised and de-identified. Patients with a definitive HBsAg immunoassay result and routine blood test values measured during pathology examination were considered. Patients with incomplete data profiles were excluded.

### 2.2. Outcome Definition and Assessment

Patients were first evaluated using routine clinical chemistry and haematology blood tests, and those suspected of HBV infection were referred for enzyme immunoassay testing. A suspected HBV case is defined as a case that was compatible with standard clinical description [20]. The primary outcome was HBV infection, assessed using HBsAg immunoassay, with results classified as either HBsAg-positive or HBsAg-negative outcome. A positive HBsAg outcome was based on the detection of HBsAg, a serological marker of infection in patient blood.

### 2.3. HepB LiveTest Model

HepB LiveTest is a machine learning model for early detection of HBV infection, translated into a publicly available web app [9]. The model was developed on the basis of 20 routine pathology attributes from 916 patients from the Nigerian Institute of Medical Research (NIMR) using cutting-edge algorithms, including an ensemble of interpretable decision trees to obtain decision thresholds to predict patient HBV infection status in real time. The model proved to be highly accurate for discriminating HBsAg-positive from negative patients (accuracy = 85.4%, sensitivity = 91%, specificity = 72.6%, precision = 88.2%, F1-score = 0.89, AUC = 0.90), with aspartate aminotransferase (AST), white blood count (WBC), age, alanine aminotransferase (ALT), and albumin as the strongest predictive markers of infection.

### 2.4. Statistics and Case-Mix Effect

Patient baseline characteristics were compared between the NIMR derivation cohort and the UITH Nigerian and SNP Australian validation cohorts. Baseline characteristics were presented as mean (±SD) for continuous variables, while categorical variables were summarised by the number of subjects (with percentages). Parametric tests were applied, since the population data have a normal distribution. The baseline characteristics between HepB LiveTest derivation cohort and the external validation cohorts were compared using the one-way ANOVA test (or Student *t*-test if two groups), and the distribution of the categorical variables was compared using Pearson’s chi-square test.

Assessing the clinical validity and generalisability of a prediction model typically involves one fundamental step, which is centred on quantifying the model’s discrimination. In this context, the discrimination measure of HepB LiveTest model would indicate the extent to which the model distinguishes between patients with and without HBV infection in the UITH-Nigerian and SNP-Australian validation cohorts. Discrimination is usually measured by the C statistic, also known as the concordance index or, for binary outcomes, the area under the receiver operating characteristic (ROC) curve [21,22]. The performance of the HepB LiveTest model on the validation cohorts was, therefore, evaluated by constructing an ROC curve and estimating the AUC (with a 95% CI) to assess the model validity across the different population settings. Delong’s method was used to calculate the 95% confidence interval (CI) of AUROC [23]. The effect of the difference in predictor values’ distribution on predictive performance was also assessed (i.e., case-mix effect). This was conducted by calculating the mean for each continuous variable in the validation cohorts and comparing with the ones in the HepB LiveTest derivation cohort. All statistical analyses were performed using R software [24]. The R source code is available online at https://github.com/bia-ml/HepB-LiveTest-validation. 

## 3. Results

Patient characteristics in the UITH-Nigerian and SNP-Australian validation cohorts in comparison with the original HepB LiveTest derivation cohort.

The final SNP-Australian sample size was 9102, while the UITH-Nigerian sample size was 258. Current evidence suggests a minimum effective sample size of 100 for external validation [25]. Patients in HepB LiveTest derivation cohort and the UITH-Nigerian and SNP-Australian validation cohorts differed in their baseline characteristics, including demographics and most pathology attributes (Table 1). Compared to the Australian validation cohort, patients in the derivation and UITH-Nigerian validation cohorts were younger (mean age, 45.5 years vs. 38.8 years vs. 40.8 years, respectively; *p* < 0.001). In addition, the SNP-Australian validation cohort had a lower baseline ALT level (57.9 U/L vs. 101 U/L vs. 182.5 U/L) and lower incidence of HBsAg positivity (1.9% vs. 69.4% vs. 57.3%) than those in the derivation and the UITH-Nigerian validation cohort, respectively. The reference interval of the pathology markers contained in the dataset are presented in Supplementary Table S1.

### 3.1. Performance of HepB LiveTest on External Patient Cohorts

**Figure 1:**
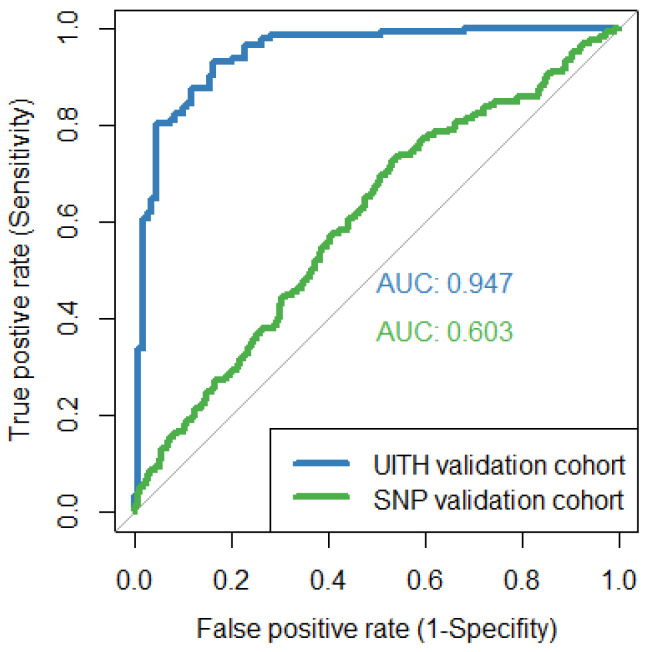
HepB LiveTest performance on UITH-Nigerian and SNP-Australian validation cohorts. The diagonal line represents the baseline that can be obtained from a random classifier and correspond to an AUC of 0.5.

The performance of HepB LiveTest is summarised into a single measure of AUC (Figure 1), as observed for each external validation cohort. Figure 1 shows that HepB LiveTest performed optimally in the UITH-Nigerian patients with an AUROC of 0.94 (95% CI, 0.91–0.97) but showed limited clinical validity in the SNP-Australian patients (0.60; 95% CI, 0.56–0.64). An AUC value near 1 means that the model has excellent discrimination, while a value close to 0.5 indicates the model discriminates no better than chance. Hence, the further the ROC curve is above the line, the better.

For the UITH-Nigerian patients, HepB LiveTest correctly identified at least 9 out of every 10 HBV patients. To put this result in context, that is an estimated 91% in sensitivity performance. However, when HepB LiveTest was tested on the SNP-Australian validation cohort, its performance dropped to 66%. The performance measures in Table 2 corroborate the findings that HepB LiveTest has adequate cross-site transportability to the UITH-based patient cohort, providing tailored predictions that ensured most cases did not go unnoticed, compared to its performance in the Australian population.

### 3.2. Inspection of Dataset Shift on Case-Mix Effect

Dataset shift in terms of the difference in the mean of predictors was observed in individual features from HepB LiveTest derivation cohort to the validation cohorts. Notable feature mean differences were found between the HepB LiveTest derivation cohort and the SNP-Australian validation cohort, and this could influence performance owing to case-mix effect. The largest differences were in GGT with 202.5% increase and ALT with −42% decrease. Many of the features between HepB LiveTest derivation cohort and the UITH-Nigerian validation cohort had similar distributions in mean, as shown in Table 3.

## 4. Discussion

We present the external validation results of HepB LiveTest, a machine learning decision support system designed for early detection of HBV using routine pathology markers. Our findings demonstrate that HepB LiveTest performs optimally in the UITH-Nigerian patient cohort but exhibits limited clinical validity in the SNP-Australian patient cohort. This suggests the need for caution when adopting the model on older populations in settings with low incidence of HBV infection and those with similar predictor value distribution observed in the Australian cohort.

The variability in prediction model performance across different settings and populations is widely recognised [17,26,27]. Therefore, conducting multiple external validation studies is crucial to fully understand the generalisability of prediction model. Various factors, including differences in outcome incidence and variations in the distribution of predictor values (i.e., case mix), can influence the heterogeneity in model performance across different settings and populations [18,19,28,29,30,31,32].

In our study, the substantial deviation in HBV incidence between the original HepB LiveTest derivation cohort and the UITH-Nigerian validation cohort (69.4% vs. 57.3%) from the low incidence observed in the Australian patient cohort (1.9%) may, in part, explain the observed performance drift. Therefore, recalibration of the model, considering changes in infection rates/outcome incidence, may be necessary when applying the model in settings with low levels of HBV infection.

The presence of heterogeneity in measurement procedures can also significantly impact the performance of prediction models [18,19,33,34]. Several factors that may contribute to this variability include variations in clinical practice patterns between clinicians and geographical locations [35,36], use of different laboratory equipment, degrees of subjectivity in measurements influenced by clinicians’ experience and backgrounds, and analytical and race-specific variability in reference intervals of blood test markers [37,38,39,40,41]. Whilst the degree of difference between measurements during model development and validation can affect the model’s discriminative performance, seemingly “better” measurements at validation, such as predictors measured under stricter protocols than in the development cohort, may also not lead to improved model performance; instead, it could even result in deteriorated performance [18,19].

In our study, we recognise that the performance drift observed in the Australian cohort compared to the Nigerian cohort may have been influenced by differences in the distribution of predictor values (i.e., case mix). The significant differences in certain feature distributions, such as GGT (202.5% increase) and ALT (−42% decrease), between the HepB LiveTest derivation cohort and Australian validation cohort may have contributed to the observed limited clinical validity. Whilst the elevated baseline serum GGT level in the Australian population might be a reflection of alcohol misuse, the normal baseline ALT level was expected for a population with low levels of HBV infection. Additionally, the lack of AST data in the Australian validation cohort, which is an important predictive marker of HBV infection required by HepB LiveTest, may have influenced the model’s performance. These findings highlight the potential impact of case-mix variability on the performance of HepB LiveTest. More broadly, the findings also suggest that multicentre external validation studies offer the potential to capture heterogeneity across different populations and settings, thus providing evidence on the appropriate level of model generalisability within specific contexts.

The incorporation of routine laboratory blood test markers in HepB LiveTest that are readily available in many outpatient and inpatient clinical settings, along with its user-friendly interface, makes it potentially deployable for early detection of HBV in Nigerian patients, without resorting to expensive second-tier immunoassay testing. However, during the clinical deployment phase, the model would need to be closely monitored for necessary updates, particularly when patient demographics and local practice patterns/norms inevitably shift. Continuous monitoring and updates will ensure that the model remains adaptable and effective in capturing evolving epidemiological trends and clinical practice patterns.

The strength of this work lies in the universal availability of the required predictive pathology markers in the majority of healthcare settings and the validation using data from external patient cohorts in two different population settings. Three studies have created a machine learning model to predict HBV infection [6,7,8]. The three studies employed a similar approach to HepB LiveTest, using a combination of simple demographic information and routine blood tests. However, models in all three studies were not translated into automated point-of-care decision-making tools for further evaluation of clinical impact and were also not externally validated.

Nonetheless, this study has limitations. It is challenging to fully understand how ethnic variability and HBV genotypic variation between Nigerian and Australian populations collectively and independently impact the performance drift of HepB LiveTest. For example, HBV genotype C is the most frequent genotype in the Australian population, while genotype E exclusively predominates Nigeria [42]. Genotype differences between populations may influence the cross-site transportability of machine learning prediction models due to biological effects [43], modified by the environment and population/genetic admixture. Further evaluations into specific ethnic and genotypic drivers will be necessary to determine what biases exist and how they can best be addressed when applying the pre-trained machine learning model to a new population setting. In addition, the performance of HepB LiveTest on HBV patients co-infected with HCV or HIV remains unknown, as the model was only trained on HBV mono-infected patients based on the available data. These aspects warrant comprehensive investigation to enhance the robustness and clinical validity of HepB LiveTest across diverse populations and patient profiles.

In conclusion, HepB LiveTest demonstrates adequate geographic validation and generalisability beyond the development cohort, with optimal performance in the hospital-based Nigerian patient cohort. Future works will be required to assess the interface integration and implementation of HepB LiveTest within the clinical workflow. It may also be necessary to evaluate the adoption of HepB LiveTest in real-world clinical settings, preferably through randomised clinical trials, to inform evidence for improved patient outcomes and process optimisation. As the first, to the best of our knowledge, externally validated machine learning decision support system for early detection of HBV, HepB LiveTest provides a platform to drive a reduction in HBV prevalence through timely linkage to care and optimise the quality of life for millions of HBV patients, particularly in underserved populations such as Nigeria. Fostering collaboration between population health scientists, clinicians and software developers will facilitate seamless integration and optimisation of HepB LiveTest into routine healthcare workflows, streamlining the clinical diagnostic process and ultimately enhancing patient outcomes.

## Figures and Tables

**Table 1:** Patient characteristics in the UITH-Nigerian and SNP-Australian validation cohorts in comparison with the original HepB LiveTest derivation cohort.

Patient Characteristics	HepB LiveTest Derivation Cohort (n = 916)	UITH-Nigerian Validation Cohort (n = 258)	SNP-Australian Validation Cohort (n = 9102)	*p*-Value
Demographics				
Age, years	38.8 ± 12.5	40.8 ± 13.5	45.5 ± 18.3	<0.001 ^a^
Sex, male, (n, %)	540 (58.9%)	154 (59.6%)	4811 (52.8%)	<0.001 ^b^
Pathology markers				
ALT, U/L	101.0 ± 225.2	182.5 ± 344.1	57.9 ± 199.3	<0.001 ^a^
AST, U/L	79.4 ± 173.7	128.4 ± 251.2	—	
ALKP, U/L	84.5 ± 40.1	85.7 ± 45.4	92.4 ± 83.1	0.008 ^a^
Crea, µmol/L	84.3 ± 48.5	81.8 ± 28.7	86.9 ± 56.2	0.148 ^a^
TBil, µmol/L	16.3 ± 35.2	18.8 ± 41.5	14.7 ± 28.3	0.029 ^a^
GGT, U/L	27.8 ± 17.5	29.2 ± 19.7	84.1 ± 213.3	<0.001 ^a^
ALB, g/L	37.2 ± 8.1	40.0 ± 6.5	43.2 ± 5.4	<0.001 ^a^
Hb, g/L	139.5 ± 19.0	137.8 ± 19.2	140.3 ± 18.0	0.046 ^a^
Hct, L/L	0.41 ± 0.05	0.4 ± 0.05	0.41 ± 0.05	0.006 ^a^
WBC, 10^9^/L	6.4 ± 3.0	6.9 ± 3.2	7.9 ± 6.5	<0.001 ^a^
PLT, 10^9^/L	252.6 ± 92.0	251.6 ± 102.9	261.9 ± 89.9	0.003 ^a^
MCHC, g/L	340.7 ± 8.1	340.4 ± 8.2	342.2 ± 7.2	<0.001 ^a^
MCH, pg/RBC	30.3 ± 2.6	30.3 ± 2.6	30.6 ± 2.2	<0.001 ^a^
MCV, fL	88.9 ± 7.0	88.9 ± 7.0	89.4 ± 5.9	0.028 ^a^
RBC, 10^12^/L	4.6 ± 0.6	4.5 ± 0.6	4.6 ± 0.6	0.030 ^a^
RDW, %	14.1 ± 2.0	14.3 ± 2.0	13.8 ± 1.6	<0.001 ^a^
Neut, %	4.96 ± 4.7	4.91 ± 2.7	4.9 ± 2.9	1.000 ^a^
Lymph, %	2.1 ± 1.0	2.1 ± 0.9	2.0 ± 1.6	0.112 ^a^
Presence of HBsAg, n (%)	636 (69.4%)	148 (57.3%)	173 (1.9%)	<0.001 ^b^

Note. Data were presented as mean ± SD for continuous variables and as number (%) for categorical variables. ALT—alanine aminotransferase; AST—aspartate aminotransferase; ALKP—alkaline phosphate; Crea—creatinine; TBil—total bilirubin; GGT—gamma glutamyl transferase; ALB—albumin; Hb—haemoglobin; Hct—haematocrit; WBC—white blood cell; PLT—platelet; MCHC—mean corpuscular haemoglobin concentration; MCH—mean corpuscular haemoglobin; MCV—mean corpuscular volume; RBC—red blood cell; RDW—red cell distribution width; Neut—neutrophils; Lymph—lymphocytes. ^a^ One-way ANOVA; ^b^ Chi-square.

**Table 2:** Other performance measures for HepB LiveTest prediction model in UITH-Nigerian and SNP-Australian validation cohorts.

HepB LiveTest Performance	Sensitivity (%)	Specificity (%)	ACC (95 CI%)
UITH-Nigerian validation cohort	91.2	83.6	87.9 (83.3–91.6)
SNP-Australian validation cohort	66.4	50.9	51.2 (50.2–52.2)

**Table 3:** Changes in mean value per clinical attribute between HepB LiveTest derivation cohort and the validation cohorts.

Clinical Attribute	Change in Mean Value %	
	NIMR-Derivation Cohort and UITH-Nigerian Validation Cohort	NIMR-Derivation Cohort and SNP-Australian Validation Cohort
Age, years	5.2	17.2
ALT, U/L	80.7	−42.7
AST, U/L	61.7	—
ALKP, U/L	1.4	9.3
Crea, µmol/L	−3.0	3.08
TBil, µmol/L	15.3	−9.8
GGT, U/L	5.0	202.5
ALB, g/L	7.5	16.1
Hb, g/L	−1.2	0.6
Hct, L/L	−2.4	0.0
WBC, 10^9^/L	7.8	23.4
PLT, 10^9^/L	−0.4	3.7
MCHC, g/L	−0.1	0.4
MCH, pg/RBC	0.0	1.0
MCV, fL	0.0	0.6
RBC, 10^12^/L	−2.2	0.0
RDW, %	1.4	−2.1
Neut, %	−1.0	−1.2
Lymph, %	0.0	−4.8

ALT—alanine aminotransferase; AST—aspartate aminotransferase; ALKP—alkaline phosphate; Crea—creatinine; TBil—total bilirubin; GGT—gamma glutamyl transferase; ALB—albumin; Hb—haemoglobin; Hct—haematocrit; WBC—white blood cell; PLT- platelet; MCHC—mean corpuscular haemoglobin concentration; MCH—mean corpuscular haemoglobin; MCV—mean corpuscular volume; RBC—red blood cell; RDW—red cell distribution width; Neut—neutrophils; Lymph—lymphocytes.

## Data Availability

The authors declare that all data supporting the findings of this study are available within the paper. Raw data are available from the corresponding author in redacted form upon reasonable request. Correspondence and requests should be addressed to B.I.A.

## Supplementary Materials

The following supporting information can be downloaded at: https://www.mdpi.com/article/10.3390/v15081735/s1, Table S1: Laboratory reference intervals for the routine pathology markers.
