# Supplementary material for: Clinical Validity of a Machine Learning Decision Support System for Early Detection of Hepatitis B Virus: A Binational External Validation Study
Source: Viruses. 2023 Aug 14;15(8):1735. doi: 10.3390/v15081735 (PMC10458613; doi:10.3390/v15081735)
Supplement: Supplementary file 1 [file viruses-15-01735-s001.zip › viruses-2535744-supplementary.pdf]

Supplementary Table

Laboratory reference intervals for the routine pathology markers

Pathology markers	Abbreviations	SNP-Australian Reference range		UITH-Nigerian reference range
		Male	Female	Male/Female
Alanine aminotransferase	ALT	5 - 40 U/L	5 - 30 U/L	10 - 49 U/L
Alkaline phosphate	ALKP	35 - 110 U/L	20 - 105 U/L	21 - 105 U/L
Creatinine	Creat	60 - 120 μmol/L	45 – 85 μmol/L	42 – 114 μmol/L
Total bilirubin	TBil	4 – 20 μmol/L	3 – 15 μmol/L	3 – 25 μmol/L
Gamma glutamyl transferase	GGT	5 - 50 U/L	5 - 35 U/L	7 - 50 U/L
Albumin	ALB	34 - 50 g/L	34 - 50 g/L	35 - 50 g/L
Haemoglobin	Hb	125 - 175 g/L	110 - 165 g/L	130 - 175 g/L
Haematocrit	Hct	0.38 - 0.54 L/L	0.34 - 0.47 L/L	0.39 - 0.48 L/L
White blood count	WBC	3.5 – 11.0 x 10^9 /L	3.5 – 11.0 x 10^9 /L	2.5 – 10.0 x 10^9 /L
Platelet	PLT	150 – 400 x 10^9 /L	150 – 400 x 10^9 /L	150 – 450 x 10^9 /L
Mean corpuscular haemoglobin concentration	MCHC	310 – 360 g/L	310 – 360 g/L	320 – 365 g/L
Mean corpuscular haemoglobin	MCH	27.5 - 34 pg/RBC	27.5 - 34 pg/RBC	27 - 34 pg/RBC
Mean corpuscular volume	MCV	80 - 100 fL	80 - 100 fL	80 - 100 fL
Red blood cell	RBC	4.2 - 6.5 x 10^{12} /L	3.7 - 5.6 x 10^{12} /L	3.9 - 5.6 x 10^{12} /L
Red cell distribution width	RDW	11-16%	11-16%	11-15%
Neutrophils	Neut	40 – 75%	40 – 75%	40 – 75%
Lymphocytes	Lymph	18 – 45%	18 – 45%	20 – 45%
Aspartate aminotransferase	AST	10 – 40%	10 – 35 U/L	10 - 46 U/L
